# Second-hand Smoke Increases Nitric Oxide and Alters the IgE Response in a Murine Model of Allergic Aspergillosis

**DOI:** 10.1080/17402520500116806

**Published:** 2005-06

**Authors:** Brian W. P. Seymour, Janice L. Peake, Kent E. Pinkerton, Viswanath P. Kurup, Laurel J. Gershwin

**Affiliations:** 1 Department of Pathology, Microbiology and Immunology School of Veterinary Medicine University of California Davis CA 95616 USA; 2 Department of Anatomy, Physiology and Cell Biology School of Veterinary Medicine University of California Davis CA 95616 USA; 3 Department of Medicine Allergy Immunology Division Medical College of Wisconsin Research Service 151-I, V.A. Medical Center Milwaukee WI 53295-1000 USA

## Abstract

This study was performed to determine the effects of environmental tobacco smoke (ETS) on nitric oxide (NO) and immunoglobulin (Ig) production in a murine model of allergic bronchopulmonary aspergillosis (ABPA). Adult BALB/c mice were exposed to aged and diluted sidestream cigarette smoke from day 0 through day 43 to simulate “second-hand smoke”. During exposure, mice were sensitized to soluble *Aspergillus fumigatus* (Af) antigen intranasally between day 14 and 24. All Af sensitized mice in ambient air (Af + AIR) made elevated levels of IgE, IgG1, IgM, IgG2a and IgA. Af sensitized mice housed in ETS (Af + ETS) made similar levels of immunoglobulins except for IgE that was significantly reduced in the serum and bronchoalveolar lavage (BAL). However, immunohistochemical evaluation of the lung revealed a marked accumulation of IgE positive cells in the lung parenchyma of these Af + ETS mice. LPS stimulation of BAL cells revealed elevated levels of NO in the Af + AIR group, which was further enhanced in the Af+ETS group. *In vitro* restimulation of the BAL cells on day 45 showed a TH0 response with elevated levels of IL3, 4, 5, 10 and IFN-γ. However, by day 28 the response shifted such that TH2 cytokines increased while IFN-γ decreased. The Af + ETS group showed markedly reduced levels in all cytokines tested, including the inflammatory cytokine IL6, when compared to the Af+AIR group. These results demonstrate that ETS affects ABPA by further enhancing the NO production and reduces the TH2 and the inflammatory cytokines while altering the pattern of IgE responses.


*Clinical & Developmental Immunology*, June 2005; 12(2): 113–124

### Second-hand smoke increases nitric oxide and alters the IgE response in a murine model of allergic aspergillosis

BRIAN W.P. SEYMOUR[1], JANICE L. PEAKE[2], KENT E. PINKERTON[2], VISWANATH P. KURUP[3], & LAUREL J. GERSHWIN[1]

[1]*Department of Pathology, Microbiology and Immunology, School of Veterinary Medicine, University of California, Davis, CA 95616, USA,* [2]*Department of Anatomy, Physiology and Cell Biology, School of Veterinary Medicine, University of California, Davis, CA 95616, USA, and* [3]*Department of Medicine, Allergy Immunology Division, Medical College of Wisconsin, Research Service 151-I, V.A. Medical Center, Milwaukee, WI 53295-1000, USA*

**Abstract**

This study was performed to determine the effects of environmental tobacco smoke (ETS) on nitric oxide (NO) and immunoglobulin (Ig) production in a murine model of allergic bronchopulmonary aspergillosis (ABPA). Adult BALB/c mice were exposed to aged and diluted sidestream cigarette smoke from day 0 through day 43 to simulate "second-hand smoke". During exposure, mice were sensitized to soluble *Aspergillus fumigatus* (Af) antigen intranasally between day 14 and 24. All Af sensitized mice in ambient air (Af + AIR) made elevated levels of IgE, IgG1, IgM, IgG2a and IgA. Af sensitized mice housed in ETS (Af + ETS) made similar levels of immunoglobulins except for IgE that was significantly reduced in the serum and bronchoalveolar lavage (BAL). However, immunohistochemical evaluation of the lung revealed a marked accumulation of IgE positive cells in the lung parenchyma of these Af + ETS mice. LPS stimulation of BAL cells revealed elevated levels of NO in the Af + AIR group, which was further enhanced in the Af + ETS group. *In vitro* restimulation of the BAL cells on day 45 showed a TH0 response with elevated levels of IL3, 4, 5, 10 and IFN-γ. However, by day 28 the response shifted such that TH2 cytokines increased while IFN-γ decreased. The Af + ETS group showed markedly reduced levels in all cytokines tested, including the inflammatory cytokine IL6, when compared to the Af + AIR group. These results demonstrate that ETS affects ABPA by further enhancing the NO production and reduces the TH2 and the inflammatory cytokines while altering the pattern of IgE responses.

**Keywords:** *Environmental tobacco smoke,* Aspergillus fumigatus*, cytokine, NO*

### Introduction

Allergic asthma has been increasing in the industrial nations (Anderson et al. 1994). This increase has stimulated epidemiologists to examine the relationship between environmental influences, atopy and asthma (Oryszczyn et al. 2000, Kuwahara et al. 2001, Simoni et al. 2001, Patino and Martinez 2001, Guilbert et al. 2004). The effect of environmental tobacco smoke (ETS) also known as 'secondhand smoke' on the pathogenesis of allergic asthma (Martinez et al. 1988, Menon et al. 1992, Gilliland et al. 2001) is under intense investigation. Epidemiological studies have implied that ETS adversely affects the health of nonsmokers. These effects range from development of cancer (Tredaniel et al. 1994) to chronic respiratory symptoms such as wheezing and chronic cough (Gilliland et al. 2001). Many of these epidemiological observations have been confirmed or are actively being investigated using animals housed in controlled smoking environments (Witschi et al. 1997).

Bronchial hyperreactivity is a key clinical sign of allergic asthma and its measurement is used as an indicator of the severity of the disease (Barnes 1989). Elevated levels of blood and tissue eosinophils, serum

Correspondence: L. J. Gershwin, D.V. M., Ph.D., Department of Pathology, Microbiology and Immunology, 1126 Haring Hall, School of Veterinary Medicine, University of California, One Shields Avenue Davis, CA. 95616. Tel: 530 752 6643. Fax: 530 752 3349. E-mail: ljgershwin@ucdavis.edu

ISSN 1740-2522 print/ISSN 1740-2530 online © 2005 Taylor & Francis Group Ltd
DOI: 10.1080/17402520500116806

IgE, increases in mucin production, thickening of the airway basement membrane and accumulation of cellular infiltrate dominated by eosinophils and mast cells are other indicators of asthma (reviewed in Berman and Weller 1992, Gern et al. 1999, Jones and Holt 2000). An increase in nitric oxide (NO) in the airway and consequently in the exhaled air of smokers with asthma has also been demonstrated in asthmatics (Horvath et al. 2004). Nitric oxide is produced by endothelial cells, airway epithelial cells and a variety of inflammatory cells in the lung (Kobzik et al. 1993, Tracey et al. 1994). The synthesis of NO is accomplished by an inducible enzyme called NO synthase (inos) (reviewed in Barnes 1995). In rats, this enzyme is produced by activated macrophages and plays a relevant role in the pathology of the disease (Kobzik et al. 1993, Hamid et al.1993).

We have previously used a murine model of allergy to show a clear association between exposure to ETS and increases in the allergic response to ovalbumin (Seymour et al. 1997, 2002). Increased levels of serum IgE, blood eosinophils and TH2 cytokines were seen in the lungs of OVA sensitized mice exposed to ETS, as compared with those sensitized to allergen and housed in filtered air. These observations prompted us to examine the effect of ETS sensitization with a more complex antigen. Therefore, in our recent study (Seymour et al. 2003) we examined the effect of ETS on an established murine model of allergic bronchopulmonary aspergillosis (ABPA) (Kurup et al. 1992). Mice were sensitized to *Aspergillus fumigatus* (Af) antigen by the intranasal route and were housed in chambers containing either aged and diluted sidestream smoke to simulate ETS or filtered air (FA) as control. Indeed, we observed and previously reported that ETS exacerbated the allergic asthma in ABPA as demonstrated by functional airway hyper-responsiveness and elevated levels of blood eosinophilia (Seymour et al. 2003). Others (Singh et al. 2003) have also demonstrated in Af sensitized mice previously exposed to mainstream cigarette smoke only in utero was sufficient to enhance bronchial hyper-responsiveness when compared to filtered air Af sensitized control mice.

In the present study, on the effects of ETS on ABPA, we performed a detailed analysis of the immunoglobulin production from the serum and the bronchoalveolar lavage (BAL) of Af sensitized mice exposed to ETS or AIR. Immunohistochemical evaluation of the lung was also performed to quantify and to examine the location of the IgE and IgG1 positive cells in the lung. Finally, in vitro stimulation of the BAL cells was done to determine the NO production and cytokine levels. We observed that ETS enhanced the NO production from LPS stimulated BAL cells. It also reduced the cytokine production in the BAL and altered the IgE response in this murine model of ABPA.

### Methods

### *Animals*

Pathogen-free female BALB/c mice from Charles Rivers (Hollister, CA) were used in this study. They were 8–9 weeks old at the start of each experiment.

### *Preparation and administration of the Af antigen*

The Af antigen was a mixture of mycelial extract and culture filtrate. Af was grown and antigen prepared as previously described (Kurup et al. 1992).

### *Research cigarettes*

The cigarette used in this study was the IR4F, which is a filtered cigarette used by research laboratories. They were obtained from the Tobacco and Health Research Institute (University of Kentucky, Lexington, KY). Once purchased, they were stored at 4°C until ready for an experiment. Two days before use, they were placed at 23°C in a chamber containing water and glycerol (this mixture was at a ratio of 0.76:0.26) in order to achieve a relative humidity of 60%.

### *The smoke generation system*

The smoke generation system was designed by Teague and colleagues (Teague et al.1994). Whenever the animals were not receiving ETS, a source of filtered air was delivered to the mice so that mice exposed to ETS could be housed in these chambers for the duration of the experiment.

### *ETS exposure*

Mice were exposed to ETS for 6 hours per day from Monday through Friday. At the end of the daily exposure, the smoke generator was turned off but the animals remained in the exposure chambers. Each exposure chamber had a measurement of 69 × 69 × 61 cm with a complete change of air every 3 min. A total of 40 mice in 8 cages were housed in a single chamber.

The total suspended particulates (TSP), relative humidity, nicotine and carbon monoxide concentrations were similar to those described previously (Seymour et al. 2003). Measurements of TSP and nicotine after the system was turned off revealed a rapid decline to nondetectable levels ($< 15\,\mu g/m^3$) during the nonsmoking period.

### *Bronchoalveolar lung lavage*

BAL cells were obtained as previously described (Medin 1976). About 10–15 lungs per group were lavaged three times with 1 ml of PBS. Approximately 2.4 ml of fluid was recovered from each lung. BAL

fluids were centrifuged and supernatants were stored at $-70°C$ until assayed for isotypes levels. BAL cells were pooled, counted and stimulated for the production of cytokines.

### *In vitro stimulation of BAL cells*

BAL cells were suspended in culture medium and stimulated at $2.5 \times 10^6$/ml in flat-bottom 24-well plates coated with anti-mouse CD3 antibody as previously described (Seymour et al. 2003). Cells were also stimulated at $2.5 \times 10^6$/ml in culture medium containing 10 μg/ml LPS from *Salmonella typhosa* (Sigma Chemical Co). Cultures were incubated at 37°C in 5% $CO_2$ for 48 h for LPS stimulation. Supernatants were harvested and stored at $-70°C$ until assayed.

### *NO measurements*

Nitric oxide production from LPS stimulated BAL cells was determined by the detection of nitrite ($NO_2^-$) concentration from the Griess reaction (Oswald et al. 1992). Briefly, 50 μl of cell supernatant was added to equal volume of Griess reagents (1.5% sulfanilamide, 0.1% napthylethelene diamine dihydrochloride, 2.5% phosphoric acid) in a microtiter plate and incubated for 10 min at room temperature in the dark. The absorbance was measured at 570 nm from an automatic microplate reader. Nitrite concentration was compared to a sodium nitrate standard curve.

### *Analysis of immunoglobulins*

Total IgE was determined using a two-step sandwich ELISA as previously described (Coffman and Carty 1986). The coating antibody, EM95, was a monoclonal anti-IgE antibody (obtained from DNAX Research Institute). The second step was a nitroiodophenyl (NIP) acetic acid conjugated rabbit anti-IgE antibody called NIP 210E. IgG1, IgM, IgG2a and IgA levels were detected using ELISA kits (Southern Biotechnology, Birmingham, AL).

### *Immunohistochemical evaluation of IgE and IgG1 positive cells in the lung parenchyma*

On day 28, lungs from 4 mice per group were inflated and fixed with 1% paraformaldehyde at 30 cm water pressure for 1 h and placed into 70% ethanol until they were processed into paraffin.

A rat anti-mouse antibody, EM95, was used to detect IgE positive cells and a goat anti-mouse IgG1 biotinylated antibody (Southern Biotechnology, Birmingham, AL.) was used to detect IgG1 positive cells in 5 μm thick paraffin sections. The sections were deparaffinized in xylene, rehydrated through a graded series of ethanol and treated with 3% hydrogen peroxide to block endogenous peroxidase activity. Nonspecific binding was blocked with 10% rabbit serum in 0.01 M PBS, pH 7.4, and sections were incubated in primary antibody diluted at 1: 10,000 (IgE) or 1: 5,000 (IgG1) in blocking serum for 1 h at 37°C. A biotinylated rabbit anti-rat, mouse adsorbed, secondary antibody was used with the IgE antibody. The Vectastain elite ABC immunoperoxidase reagents (Vector Laboratories, Burlingame, CA) were used according to manufacturer's recommendation. 3,3′-Diaminobenzidine (DAB) tetrahydrochloride tablets (Sigma, St. Louis, MO) mixed with nickel chloride were used as the peroxidase substrate followed by a nuclear fast red test counterstain. Lung tissue from female BALB/c mice infected with *Nippostrongylus brasiliensis,* 12 days post infection, was used as a positive tissue control for IgE. The negative tissue control was taken from BALB/c IL4 deficient mice that cannot produce IgE. For a negative reagent control, the primary antibody was substituted with rat serum and used on experimental tissue. The negative controls did not show positive staining for IgE while the positive control did show IgE positive cells. The negative reagent control for IgG1 was normal goat serum at 1:5000.

### *Morphometric analysis of IgE and IgG1 positive cells*

To determine the number of IgE and IgG1 positive cells present in the lung parenchyma, including blood vessels, up to 20 random nonoverlapping fields were counted per animal ($n = 4$). Each field could contain parenchymal tissues as well as blood vessels. Only fields containing large airways were excluded. About 20 fields were counted for IgE and 10 for IgG1. Counts were performed using the hidden line exclusion principle (Gundersen 1977, Weibel 1979), i.e. only IgE or IgG1-positive cell profiles completely in the field were counted as well as cell profiles touching the left-hand or upper borders of the field, but not the right-hand or lower borders of the field. Only cells with abundant amounts of cytoplasm (suggestive of plasma cells) strongly staining for IgE or IgG1 were counted, although numerous positive cells with thin rims of cytoplasm (suggestive of B-lymphocytes) strongly staining for IgE and IgG1 were also observed in tissue sections.

### *Cytokine ELISA*

Sandwich ELISAs were done to measure IL3, IL5, IL6, IL10 and IFN-γ as previously described (Abrams 1995).

### *Analysis of IL4*

IL-4 from cultured supernatant was detected by a bioassay using the IL4 dependent, CT.4S cell line (kindly donated by Dr. William Paul, NIH) as previously described (Seymour et al. 1997).

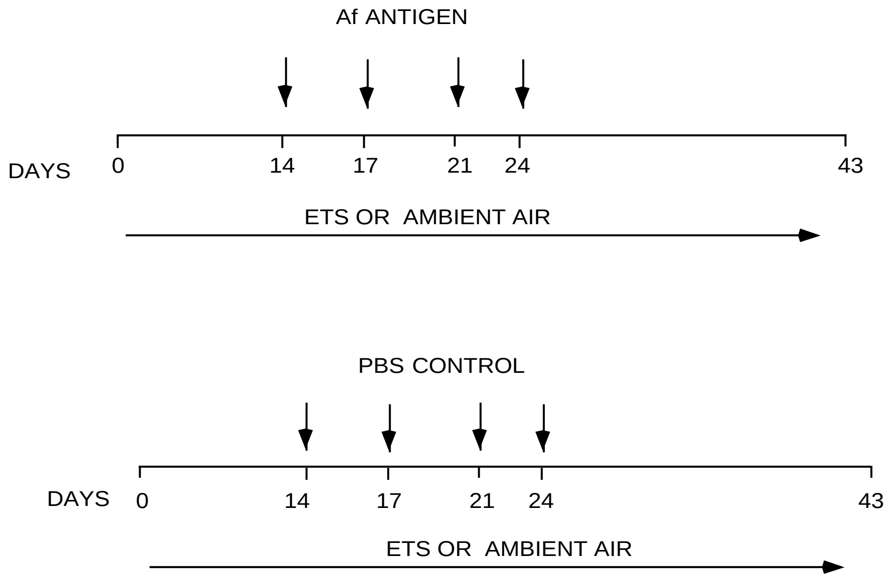

Figure 1. Antigen sensitization and ETS/AIR exposure protocols.

*Statistics*

Levels of antibodies, eosinophils and cytokines were calculated as mean and standard error of the mean. The two-tailed $p$ values were calculated according to the Mann-Whitney Test. A value of $p < 0.05$ was considered significant. Statistics for the morphometric analysis of IgE and IgG1 positive cells were calculated using one-way analysis of variance and Fisher's PLSD, Scheffe and Bonferroni/Dunn tests (Statview 4.5, Abacus Concepts, Inc., Berkeley, CA). These statistical procedures permitted comparison between PBS control groups and the corresponding various doses of Af antigen groups as well as between air and ETS groups. Significance was considered at a $p$-value of $<0.05$.

### Results

*Experimental protocol*

BALB/c mice were exposed to ETS or AIR from day 0 to 43. They were sensitized by installation of 50 µl of soluble Af antigen into their nostrils on days 14, 17, 21 and 24 (Figure 1). Four different doses of Af were used in this study 200, 100, 50 and 25 µg. Control mice were given 50 µl of PBS i.n. and exposed either to ETS or ambient air from days 0 to 43. The ETS concentration as described in the Materials and Methods was at a high ambient level, but similar to those observed in restaurants, bars and the homes of smokers. During ETS/AIR exposures animals were bled from their tail veins for IgE, IgG1 antibodies determination. Cytokine production was carried out by in vitro stimulation of BAL cells after the Af or PBS challenges.

*NO production from LPS stimulated BAL cells and its effect on IL6 production*

To test the activated state of alveolar macrophages, BAL cells were stimulated with LPS for $NO_2^-$ and the production of the inflammatory cytokine, IL6. $NO_2^-$ was substantially elevated in groups exposed to Af compared to the PBS control groups (Figure 2A). However, ETS influenced the $NO_2^-$ release as BAL cells from the Af + ETS group made 47% more $NO_2^-$ when compared to the Af + AIR group (18.93 vs. 12.98 µM on day 25 and 39.50 vs. 26.82 µM on day 28, respectively). This percentage difference was observed in 2 of 3 experiments as the third experiment revealed 100% more $NO_2^-$ from the Af + ETS group when compared to the Af + AIR group. Endogenous production of $NO_2^-$ was not detected from the BAL cultures incubated in medium without LPS.

To evaluate the effect of NO on the inflammatory cytokine production, the supernatant from the LPS stimulated BAL cells was tested for the production of IL6. All groups made a substantial production of IL6 (Figure 2B). However, we observed that the 47% increase in $NO_2^-$ from the Af + ETS group over the Af + AIR group was associated with a decrease of the same percent in IL6 in the Af + ETS group when compared to the Af + AIR group demonstrating an inverse relationship between $NO_2^-$ and IL6.

*Antibody levels in the serum and BAL of Af sensitized mice exposed to ETS*

Mice exposed to Af antigen made elevated levels of serum IgE (Figure 3). At peak response, there was approximately a 30-fold increase in IgE from the

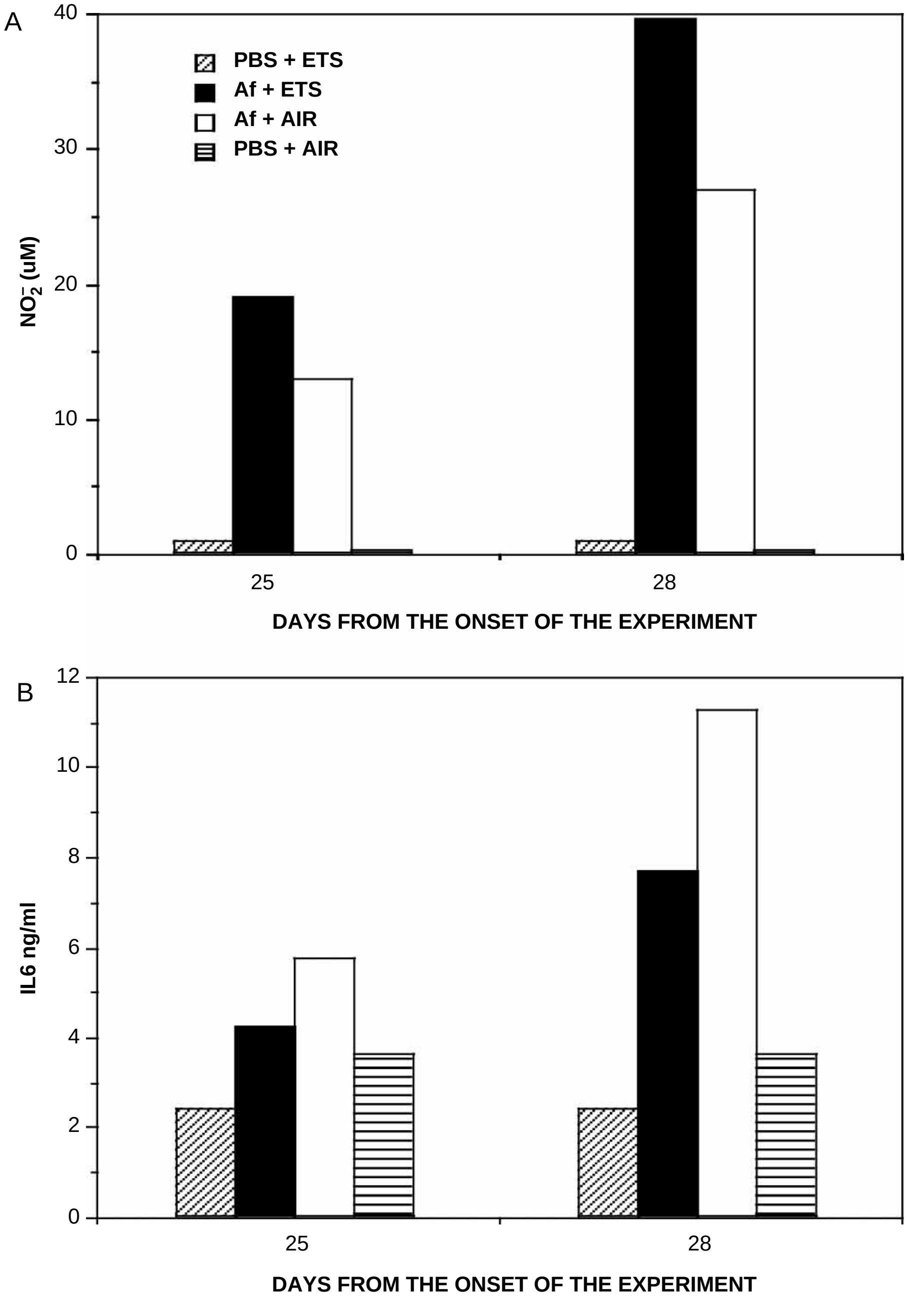

Figure 2. LPS stimulation of BAL cells for the production of $NO_2^-$ and IL6. BALB/c mice were sensitized with 200 µl Af / exposure and BAL cells were obtained on days indicated on graph. Cells were stimulated with 10 µg of LPS / ml. as described in the materials and methods. Supernatants were harvested at 48 h and assayed for $NO_2^-$ (A) and IL6 (B) production. Results are from pooled BAL cells of 15 mice / group at each timepoint.

Af + AIR group that received 200 µg Af antigen (Figure 3A). IgE levels in this group remained steady for 2 weeks after the last Af challenge. However, IgE levels in the 200 µg Af + ETS group were reduced when compared to the Af + AIR group. At day 29 (5 days after the last Af challenge), the total IgE in the serum of Af + ETS mice was 13,914 ± 3364 ng/ml compared to 21,079 ± 4995 ng/ml in the Af + AIR group. This reduction of IgE was most significant at day 44. Here, the levels of IgE in the Af + ETS group was 8859 ± 3057 ng/ml compared to 25419 ± 4976 ng/ml ($p = 0.0148$; $n = 8$) in the Af + AIR group. Elevation of IgE was also observed from mice that received 100 µg of Af i.n (Figure 2B). However, there was a rapid decline of IgE levels in both groups on day 34 (10 days after the last antigen challenge). Significant reduction of IgE was also observed in the Af + ETS group when compared to the Af + AIR group. The most significant reduction of IgE was seen at peak response on day 27

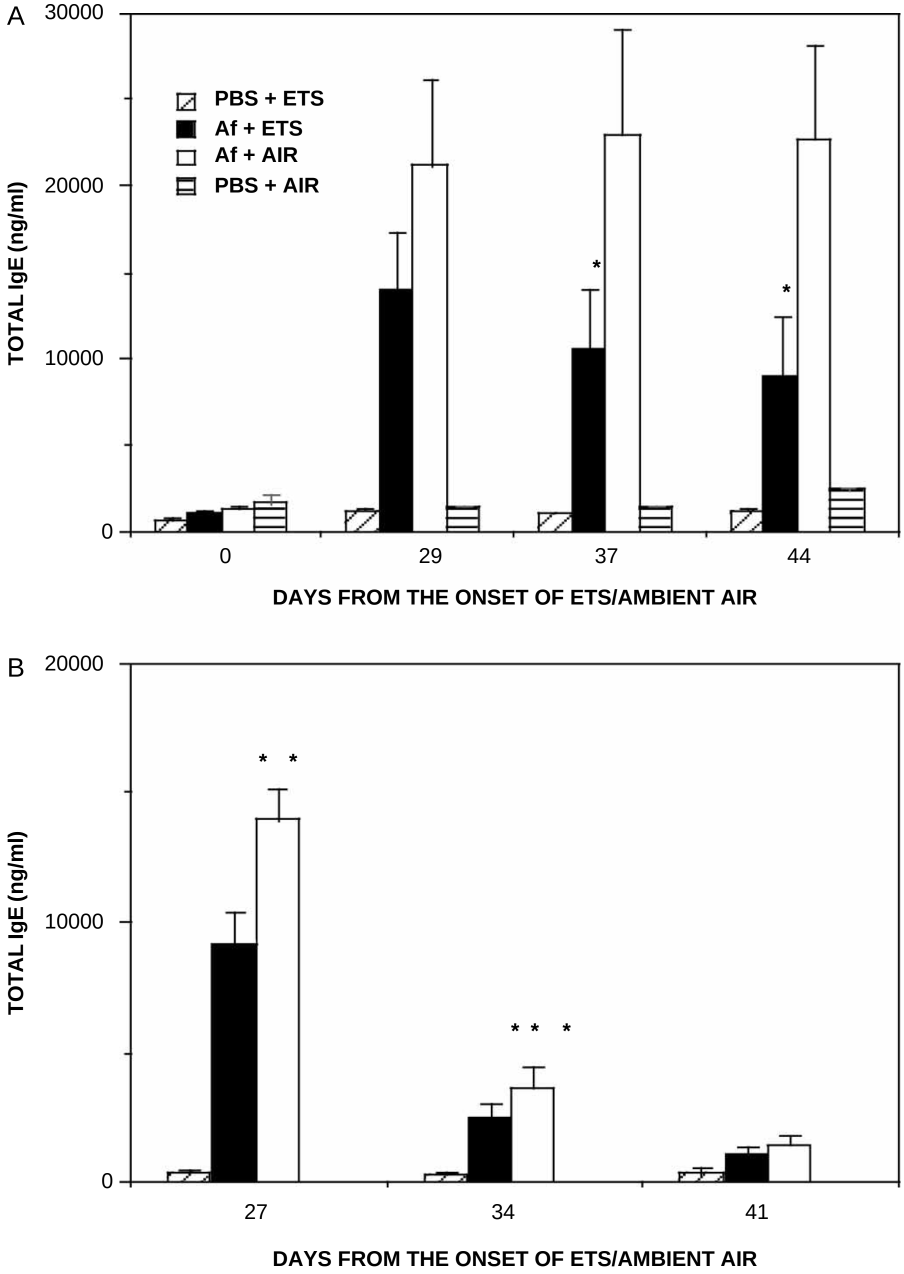

Figure 3. Total serum IgE in nanograms per milliliter estimated by ELISA in the four groups of BALB/c mice exposed to ETS or ambient air. Sensitization and exposure protocols were described in Figure 1. BALB/c mice were sensitized i.n. with either 200 μg Af per challenge (A) or 100 μg Af per challenge (B). * indicates $p < 0.05$ vs. Af + AIR group ($n = 10$). ** indicates $p = 0.0078$ vs. Af + ETS group ($n = 23$). ***indicates $p = 0.04$ vs. Af + ETS group ($n = 23$).

($p = 0.0078$; $n = 23$). We were unable to detect any significant differences in IgE levels between the PBS control groups.

There was a 13-fold increase in total IgG1 in mice challenged with 200 μg of Af (Figure 4A). However, we observed only a slight increase in this antibody when mice were challenged with 100 μg Af (Figure 4B). We were unable to detect any significant differences in total IgG1 in the Af + ETS group when compared to the Af + AIR group as both groups made comparable levels of total serum IgG1. Likewise, the PBS + ETS animals showed no changes in total IgG1 from the PBS + AIR controls.

Examination of the BAL showed a significant enhancement of all isotypes tested in Af sensitized mice when compared to the PBS controls (Table I). However, a significant reduction of total IgE in the Af + ETS group was seen at day 28 when compared to the Af + AIR group (432 ± 75 ng in the Af + AIR group vs. 210 ± 45 ng from the Af + ETS group; $p = 0.028$, $n = 15$).

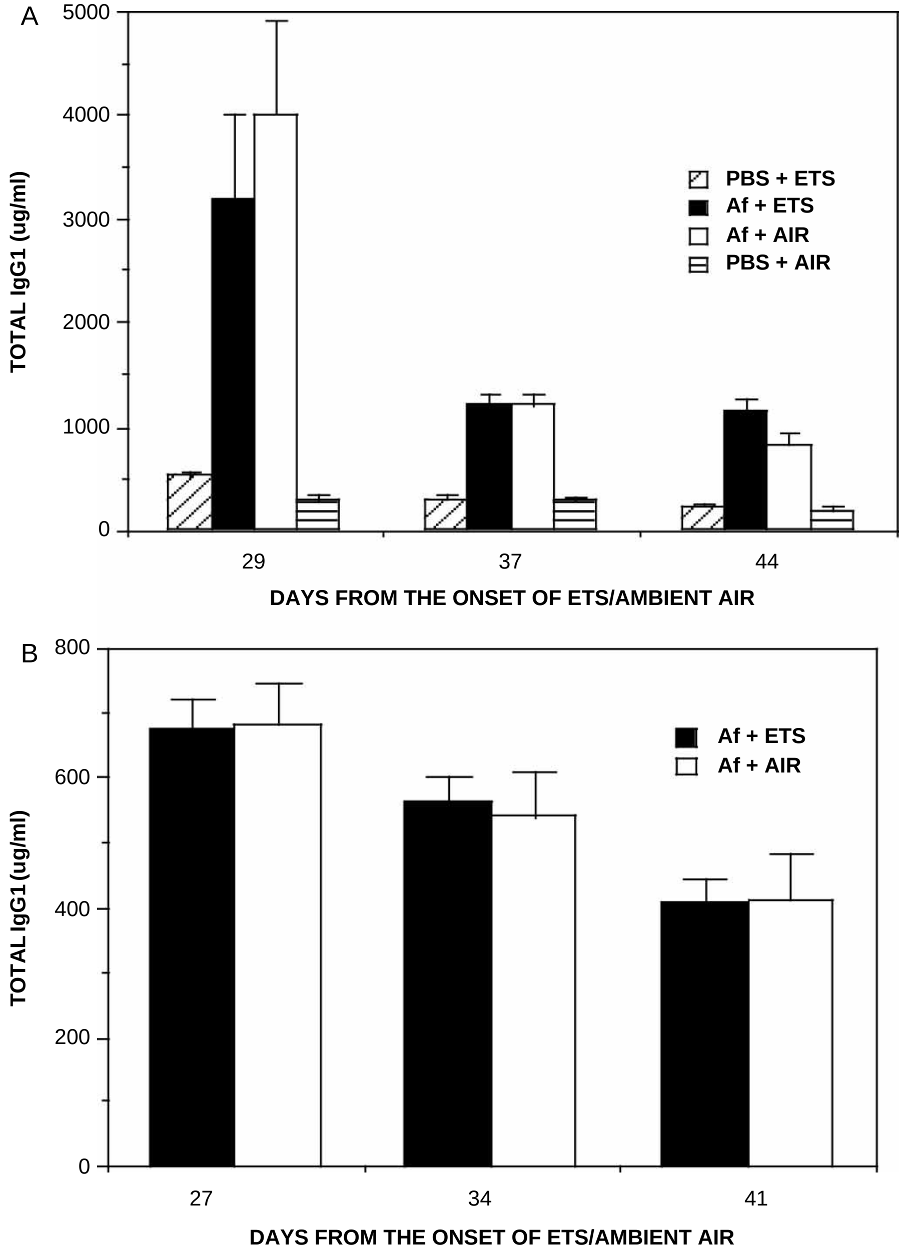

Figure 4. Total serum IgG1 in micrograms per milliliter estimated by ELISA in BALB/c mice exposed to ETS or ambient air. Sensitization and exposure protocols were described in Figure 1. BALB/c mice were sensitized i.n. with either 200 μg Af per challenge (A) or 100 μg Af per challenge (B).

*Immunohistochemical and morphometric evaluation of IgE and IgG1 positive cells in the lung parenchyma of Af sensitized mice exposed to ETS*

Immunohistochemical and morphometric analysis was used to examine the frequency of IgE positive cells within the lung parenchyma as well as the surrounding blood vessels within perivascular cuffs. Figure 5A illustrates the parenchyma and blood vessel of a normal PBS animal while Figure 5B–F illustrates animals that have been sensitized with Af antigen displaying prominent perivascular infiltration of mononuclear cells. Numerous cells were found within this influx of mononuclear cells with IgE positive staining cytoplasm. These cells, identified as plasma cells, showed a significant increase in number in the high dose Af + ETS group compared to the PBS + AIR controls, the PBS + ETS exposed animals, and both the low dose Af exposed groups. Although there was a notable increase in the number of IgE positive cells in the high dose Af + ETS group compared to high dose Af + Air group, it did not reach a level of statistical significance. Another subpopulation of mononuclear cells with very thin cytoplasmic rims staining for IgE, identified as B-lymphocytes, were observed in areas of intense cellular influx particularly

Table I. Total immunoglobulin levels in the BAL of Af sensitized and control mice.

| SENSITIZATION (i.n.) | EXPOSURE | IgE ng/ml | IgG1 ng/ml | IgM ng/ml | IgG2a ng/ml | IgA ng/ml |
|---|---|---|---|---|---|---|
| | | | DAY 25 | | | |
| PBS | AIR | <3.75 | 1874 ± 612 | 438 ± 162 | 1047 ± 324 | 276 ± 72 |
| PBS | ETS | <3.75 | 1752 ± 720 | 303 ± 78 | 741 ± 198 | 345 ± 153 |
| 200 μg Af /exposure | AIR | 32 ± 3 | 20799 ± 2463 | 4911 ± 717 | 3011 ± 582 | 3084 ± 522 |
| 200 μg Af /exposure | ETS | 32 ± 5 | 18018 ± 3201 | 3363 ± 393 | 2430 ± 399 | 2625 ± 378 |
| | | | DAY 28 | | | |
| PBS | AIR | <3.75 | 447 ± 54 | 66 ± 36 | 349 ± 53 | 195 ± 21 |
| PBS | ETS | <3.75 | 456 ± 24 | 57 ± 18 | 291 ± 34 | 102 ± 9 |
| 200 μg Af /exposure | AIR | 432 ± 75* | 22343 ± 3210 | 7689 ± 1095 | 5247 ± 469 | 1991 ± 954 |
| 200 μg Af /exposure | ETS | 210 ± 45* | 22863 ± 2364 | 5514 ± 651 | 5496 ± 533 | 2857 ± 1005 |

BAL samples were obtained from 12-15 mice/group at each timepoint and assayed for antibody titers by ELISA.
*$p = 0.028$.

around blood vessels throughout the lungs, but were not quantitated. Immunohistochemical and morphometric analysis was also applied for examining the distribution of IgG1 positive cells within these same regions. Figure 5H illustrates the results of this morphometric analysis while Figures 5E and F illustrate the distribution and appearance of IgG1 positive cells within the lung parenchyma. Although a notable increase in IgG1 positive cells were noted in animals receiving a high dose of Af antigen followed by exposure to ETS, this difference was not statistically significant when compared to the Af + AIR group.

*Cytokine production by BAL cells*

We studied the cells recruited to the lungs by examining the cytokine profile of the BAL cells at days 25 and 28 (Table II). At day 25 (24 h after the last administration of Af), there was an increase in IL3, IL4, IL5, IL10 and IFN-γ in Af sensitized groups when compared to the PBS control groups showing a Th0 cytokine profile. However, by day 28 the levels of IFN-γ decreased while the Th2 cytokines remained elevated in Af sensitized mice. Furthermore, stimulation of BAL cells revealed reduced levels of cytokines in the Af + ETS group when compared to the Af + AIR group.

### Discussion

Inhalation of cigarette smoke has been shown to have a wide range of immunological effects ranging from enhanced humoral responses (El–Nawawy et al.1996, Ronchetti et al. 1992) to suppression of the immune system (Sopori 2002). Enhanced humoral responses such as allergic sensitization, occurs from low exposure to cigarette smoke (reviewed in Holt 1987) while immunosuppression occurs from chronic exposure to high doses of mainstream smoke (Sopori et al. 1993). This immunosuppression, as demonstrated in rats, is due to the high levels of nicotine in mainstream smoke (Sopori et al. 1993). ETS contains at least 10 fold less nicotine than mainstream smoke (Seymour et al. 1997). These low doses of nicotine have been shown to be ineffective in causing suppression of the immune response (Geng et al. 1995, Geng et al. 1996).

Previously, we have shown that ETS causes exacerbation of asthma as demonstrated by functional airway hyperreactivity and elevated levels of blood eosinophilia in Af sensitized mice (Seymour et al. 2003). In our present study, we observed that the Af + ETS mice made reduced levels of IgE in the blood and BAL when compared to the Af + AIR group. In humans, the IgE response is suppressed in chronic smokers (>20 cigarette / day) but is elevated in mild smokers (reviewed in Holt 1987) and individuals exposed to ETS (El-Nawawy et al.1996, Ronchetti et al. 1992). However, biphasic changes have not been observed in smokers exposed to fungal antigens as investigators have shown that antibodies were always lower in smokers than in nonsmokers. For example, in a large survey of farmer's lung disease, in which 1444 Canadian farm workers were tested for Af and *Saccharopolyspora rectivirgula* antigens, there were 8 times more precipitating antibodies to these microbial antigens in nonsmokers when compared to smokers (Gruchow et al. 1981). In other studies to detect serum antibodies to pigeon serum antigens, there were 18.6% vs. 50.5% (McSharry et al. 1984) and 4.3% vs. 55.4% (McSharry et al. 1985) in smokers versus nonsmokers, respectively. Though we were unable to detect differences in serum IgG1, the reduced levels of the BAL and serum IgE response in this study may reflect similar occurrences for nonsmokers exposed to ETS and fungal antigens.

Exposure to Af results in both an immunological and an inflammatory response, in which the first line of defense involves the mucus epithelial barrier and the alveolar macrophage (Kauffman et al. 1995). Investigators have shown that despite the decreased level of phagocytosis of bacteria and inert particles from smoke exposed alveolar macrophages their ability to take up fungal antigens is normal

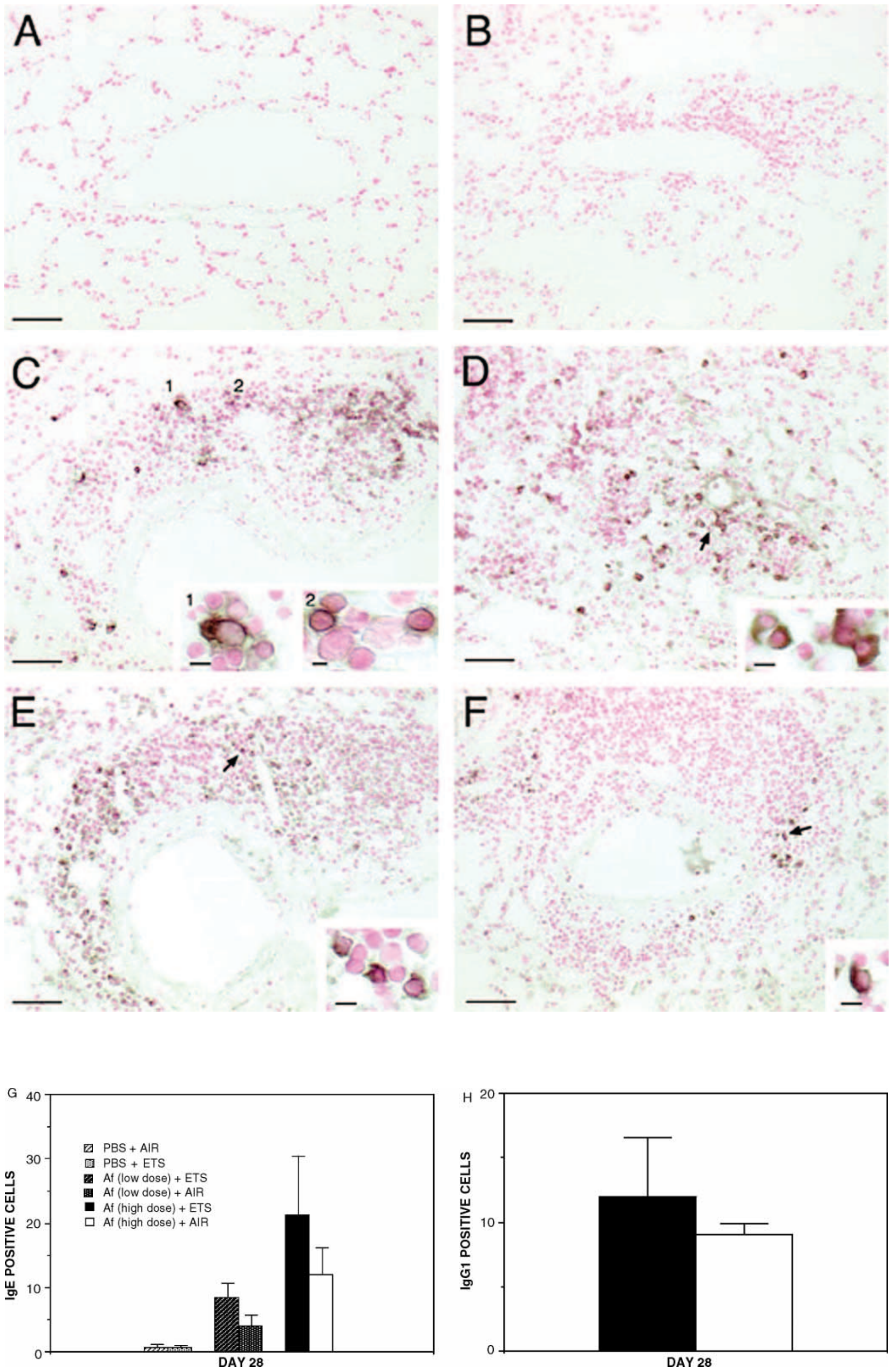

Figure 5. IgE and IgG1 immunohistochemical staining of lung tissue in paraffin sections. Mice were exposed to either 200 μg Af (high dose) or 50 μg Af (low dose) per exposure or PBS as described in Figure 1 and lungs removed and processed as described in the Methods section. Normal blood vessels in a PBS + AIR treated mouse stained with IgE antibody (panel A). Blood vessel with mononuclear cells in the perivascular interstitial space from the Af(high dose) + ETS group (panel B). This section is a rat serum negative control for IgE antibody localization. No positive staining is evident. Blood vessel from Af(high dose) + AIR (panel C) and Af(high dose) + ETS group (panel D) showing a prominent influx of cells within the perivascular cuff containing numerous IgE positive cells. The inset in panel C-1 and D, (bar = 5 μm), illustrate cells with strong cytoplasmic staining for IgE, suggestive of plasma cells. The inset in panel C-2 (bar = 2 μm) illustrates a second type of mononuclear cell with a thin rim of cytoplasm staining for IgE, suggestive of B lymphocytes. Blood vessels from Af(high dose) + AIR (panel E) and Af(high dose) + ETS group (panel F) with numerous cells in the perivascular cuff staining for IgG1. The inset in panels E and F, (bar = 5 μm), show cytoplasmic staining for IgG1. For panels A–F the scale bar = 50 μm. (G) is the number of IgE positive cells in 20 random nonoverlapping fields per animal and (H) is the number of IgG1 positive cells in 10 fields per animal ($n = 4$). Quantification is described in the methods section.

Table II. Cytokines produced after anti-CD3 stimulation of BAL cells.

| SENSITIZATION (i.n.) | EXPOSURE | IL3 ng/ml | IL4 ng/ml | IL5 ng/ml | IL10 u/ml | IFN-γ ng/ml |
|---|---|---|---|---|---|---|
| | | | DAY 25 | | | |
| PBS | AIR | 0.36 | 0.22 | <0.16 | <1.25 | 1.24 |
| PBS | ETS | 0.24 | 0.19 | <0.16 | <1.25 | 0.79 |
| 200 μg Af /exposure | AIR | 9.00 | 2.17 | 3.51 | 61.90 | 6.00 |
| 200 μg Af /exposure | ETS | 6.60 | 1.71 | 2.11 | 61.30 | 7.00 |
| | | | DAY 28 | | | |
| PBS | AIR | 0.47 | <0.08 | <0.16 | <1.25 | 0.39 |
| PBS | ETS | 0.17 | <0.08 | <0.16 | <1.25 | <0.16 |
| 200 μg Af /exposure | AIR | 18.05 | 6.52 | 8.37 | 84.27 | 3.30 |
| 200 μg Af /exposure | ETS | 9.81 | 3.17 | 3.86 | 34.47 | 1.40 |

In vitro stimulation was performed on pooled Bal cells from15 mice/group on day 25 and 28 as described in the methods section.

(Harris et al. 1970, Mann et al. 1971). The BAL of humans who smoke show a three to five fold increase in alveolar macrophages (reviewed in Holt 1987). Similar expansion of these cells was demonstrated in mice exposed to ETS (Seymour et al. 1997). Furthermore, the smoke-exposed alveolar macrophage contains increased endoplasmic reticulum, ribosomes, large lysosomes and cytoplasmic inclusions suggesting that they are activated in vivo (Holt 1987). Alveolar macrophages have been shown to be suppressive to the immune response (Holt et al. 1993). Thus, these increased levels of alveolar macrophages coupled with their activated state and ability to take up fungal antigens may have made them more efficient in the processing of the Af antigens, which may explain the reduced levels of IgE in the Af + ETS group when compared to the Af + AIR group.

In order to understand the mode of action of the alveolar macrophage in causing IgE suppression, we examined the level of NO from BAL cells after ETS exposure. Many studies have associated the expression of NO with patients suffering from asthma (Hamid et al. 1993, Oh et al. 2003, Mahut et al. 2004) and that its expression is elevated in cigarette smokers with this disease (Jang et al. 2002). However, studies have also shown that activated macrophages are capable of releasing NO that is involved in suppressing an immune response (Al-Ramadi et al. 1992). Studies in rats (Meldrum et al. 1998) and humans (Chollet-Martin et al. 1996, Thomassen et al. 1997) have implicated this molecule with the inhibition of inflammatory cytokines. We have seen enhanced levels of $NO_2^-$ from the LPS stimulated BAL of ETS exposed mice and decrease in cytokines when these cells were restimulated in vitro. In our previous study, we were unable to see the differences in the cytokine levels between the groups when we stimulated the homogenized lung cells in vitro (Seymour et al. 2003). Thus, we examined the BAL since we believed it would offer a more accurate representation of the immunological status of the mice from exposure to Af and ETS. We suggest that the enhanced levels of $NO_2^-$ may be partly responsible for the decrease in cytokines and circulating IgE in the Af + ETS group. We were unable to see significant enhancement of $NO_2^-$ in OVA sensitized mice as this may explain the absence of IgE suppression in OVA sensitized adult mice exposed to ETS (unpublished data).

Despite the reduced levels of IgE in the BAL and serum of Af + ETS mice, immunohistochemical staining for cell-associated IgE in the lung parenchyma revealed more IgE positive stained cells from the Af + ETS group when compared to the Af + AIR group. This alteration in the immunoglobulin response was isotype specific as no differences were observed in numbers of IgG1 staining cells between animals exposed to ETS or air. This data, while possibly having biological significance, did not attain a level of statistical significance.

The differences in secreted IgE levels in BAL and serum vs. numbers of IgE containing plasma and/or B cells in the lung may be attributed to sampling error. Alternatively, differences in regulation of IgE at the transcriptional level may account for this data. Studies have shown that RNA isolated from human and mouse IgE secreting cells contain a series of alternatively spliced epsilon (ε) mRNA with some corresponding to membrane bound IgE and others to its secreted form (Zhang et al. 1992, Saxon et al. 1995, Hellman 1993). Expression of these alternatively spliced ε-mRNA have been shown to be differentially regulated in humans (Diaz-Sanchez et al. 1995). For example, Fc ε RII crosslinking of human B cells results in suppression of an ongoing IgE response with decrease in ε mRNA for secreted but not membrane bound IgE (Saxon et al. 1991). Therefore, it is possible that similar types of regulation may be occurring at the transcriptional level that resulted in the alteration of IgE responses in the Af + ETS group.

Saxon and colleagues have shown that inhalation of diesel exhaust particles resulted in alteration of the relative ratios of membrane bound to secreted isoforms of ε mRNAs that resulted in increases in IgE secreting cells (Diaz-Sanchez et al. 1994). This increase in IgE secreting cells was later shown to be due to polycyclic aromatic hydrocarbons (PAH) present in diesel exhaust particles (Takenaka et al. 1995). This molecule is also a constituent of cigarette smoke (Scherer and Richter 1997) and has been shown to induce cytochrome P4501A1, an isoenzyme elevated in the lung of cancer patients (reviewed in Gebremichael et al. 1996). Indeed, the quantity of ETS delivered to mice in our study contains PAH capable of inducing this isoenzyme. PAH along with a variety of stimuli can alter the ε mRNA splice patterns differently, and different disease states have different epsilon splice patterns (discussed in Diaz-Sanchez et al. 1994). Therefore, it is also possible that the combination of PAH from ETS with Af may also uniquely alter the pattern of the IgE response. This combination may cause a preferential decrease in mRNA coding for secreted IgE with upregulation of mRNA for its membrane bound isoform, which may explain the reduction of secreted IgE and enhancement of its membrane bound form.

In summary, this model shows that ETS increases the likelihood of allergic asthma and alters the pattern of IgE responses in Af sensitized mice. There is a reduction of secreted IgE, in the presence of increased numbers of cells either with membrane-bound or cytoplasmic IgE in the lung. This data is in contrast to our previous studies using an ovalbumin sensitized model. Our previous findings have shown that this level of ETS caused significant elevation in serum IgE and IgG1 in OVA sensitized mice when compared to those in ambient air (Seymour et al. 1997). Thus, the physical characteristics of the antigen along with the concentration of tobacco smoke may have a major role in determining the outcome of the immune response.

### Acknowledgements

The authors thank Kathleen E. Friebertshauser, Michael Goldsmith and Steve Teague for their technical assistance with the cigarette smoke generator system and the excellent care of the animals while they were housed in the exposure chambers. Animals were cared for under NIH guidelines. Animal care protocol was approved by the University of California, Davis Committee on Animal Use and Care.

We acknowledge Stuart Robinson for technical assistance and Dr. Robert Coffman for expert advice in the preparation of this manuscript.

This research was supported in part by the University of California, Tobacco-Related Disease Research Program. This work was partially supported by a US Veterans Affairs Medical research grant.

### References

Abrams J. 1995. Immunoenzymetric assay of mouse and human cytokines using NIP-labelled anti-cytokine antibodies. In: Roico R, editor. Current protocol in immunology. vol. 6. New York: 6.20.1 John Wiley and sons, Inc.

Al-Ramadi BK, Meissler Jr., JJ, Huang D, Eisenstein TK. 1992. Immunosuppression induced by nitric oxide and its inhibition by interleukin-4. Eur J Immunol 22:2249–2254.

Anderson HR, Butland BK, Strachan DP. 1994. Trends in prevalence and severity of childhood asthma. Br Med J 308:1584–1585.

Barnes PJ. 1989. New concepts in the pathogenesis of bronchial hyperresponsiveness and asthma. J Allergy Clin Immunol 83:1013–1026.

Barnes PJ. 1995. Nitric oxide and airway disease. Ann Med 27:389–393.

Berman JS, Weller PF. 1992. Airway eosinophils and lymphocytes in asthma Birds of a feather? Am Rev Respir Dis 145:1246–1248.

Chollet-Martin S, Gatecel C, Kermarrec N, Gougerot-Pocidalo M, Payen DM. 1996. Alveolar neutrophil functions and cytokine levels in patients with the adult respiratory distress syndrome during nitric oxide inhalation. Am J Respir Crit Care Med 153:985–990.

Coffman RL, Carty J. 1986. A T cell activity that enhances polyclonal IgE production and its inhibition by interferon-gamma. J Immunol 136:949–954.

Diaz-Sanchez D, Dotson AR, Takenaka H, Saxon A. 1994. Diesel exhaust particles induce local IgE production *in vivo* and alter the pattern of IgE messenger RNA isoforms. J Clin Investig 94:1417–1425.

Diaz-Sanchez D, Zhang K, Nutman TB, Saxon A. 1995. Differential regulation of alternative 3′ splicing of epsilon messenger RNA variants. J Immunol 155:1930–1941.

El-Nawawy A, *et. al.* 1996. Effect of passive smoking on frequency of respiratory illnesses and serum immunoglobulin-E (IgE) and interleuken-4 (IL-4) concentrations in exposed children. J Trop Pediatr 42:166–168.

Gebremichael A, Tullis K, Denison MS, Cheek JM, Pinkerton KE. 1996. Ah-receptor-dependent modulation of gene expression by aged and diluted sidestream cigarette smoke. Toxicol Appl Pharmacol 141:76–83.

Geng Y, Savage SM, Johnson LJ, Seagrave J, Sopori ML. 1995. Effects of nicotine on the immune response. I. Chronic exposure to nicotine impairs antigen receptor-mediated signal transduction in lymphocytes. Toxicol Appl Pharmacol 135:268–278.

Geng Y, Savage SM, Razani-Boroujerdi S, Sopori ML. 1996. Effects of nicotine on the immune response II. Chronic nicotine treatment induces T cell anergy. J Immunol 156:2384–2390.

Gern JE, Lemanske RF, Jr, Busse WW. 1999. Early life origins of asthma. J Clin Investig 104:837–843.

Gilliland FD, Li YF, Peters JM. 2001. Effects of maternal smoking during pregnancy and environmental tobacco smoke on asthma and wheezing in children. Am J Respir Crit Care Med 163: 429–436.

Gruchow HW, Hoffmann RG, Marx jr, JJ, Emanuel DA, Rimm AA. 1981. Precipitating antibodies to farmer's lung antigens in a Wisconsin farming population. Am Rev Respir Dis 124:411–415.

Guilbert TW, et al. 2004. Atopic characteristics of children with recurrent wheezing at high risk for the development of childhood asthma. J Allergy Clin Immunol 114:1282–1287.

Gundersen HJG. 1977. Notes on the estimation of the numerical density of arbitrary profiles: The edge effect. J Microsc 111: 219–223.

Hamid Q, et al. 1993. Induction of nitric oxide synthase in asthma. Lancet 342:1510–1513.

Harris JO, Swenson EW, Johnson JE. 1970. Human alveolar macrophages: Comparison of phagocytic ability, glucose utilization, and ultrastructure in smokers and nonsmokers. J Clin Investig 49:2086–2096.

Hellman L. 1993. Characterization of four novel e chain mRNA and a comparative analysis of genes for immunoglobulin E in rodents and man. Eur J Immunol 23:159–167.

Holt PG, et al. 1987. Immune and inflammatory function in cigarette smokers. Thorax 42:241–249.

Holt PG, et al. 1993. Downregulation of the antigen presenting cell function(s) of pulmonary dendritic cells in vivo by resident alveolar macrophages. J Exp Med 177:397–407.

Horvath I, Donnelly LE, Kiss A, Balint B, Kharitonov SA, Barnes PJ. 2004. Exhaled nitric oxide and hydrogen peroxide concentrations in asthmatic smokers. Respiration 71: 463–468.

Jang AS, Choi IS, Jeong TK, Lee KY. 2002. The effect of cigarette smoking on the levels of nitric oxide metabolites in the sputum of patients with acute asthma. J Asthma 39:211–216.

Jones CA, Holt PG. 2000. Immunopathology of allergy and asthma in childhood. Am J Respir Crit Care Med 162:S36–S39.

Kauffman HF, Tomee JFC, van der Werf TS, de Monchy JGR, Koeter GK. 1995. Review of fungus-induced asthmatic reactions. Am J Respir Crit Care Med 151:2109–2115, discussion 2116.

Kobzik L, Bredt DS, Lowenstein CJ, Drazen J, Gaston B, Sugarbaker D, Stamler JS. 1993. Nitric oxide synthase in human and rat lung: Immunocytochemical and histochemical localization. Am J Respir Cell Mol Biol 9:371–377.

Kurup VP, Mauze S, Choi H, Seymour BWP, Coffman RL. 1992. A murine model of allergic bronchopulmonary aspergillosis with elevated eosinophils and IgE. J Immunol 148:3783–3788.

Kuwahara Y, Kondoh J, Tatara K, Azuma E, Nakajima T, Hashimoto M, Komachi Y. 2001. Involvement of urban living environments in atopy and enhanced eosinophil activity: Potential risk factors of airway allergic symptoms. Allergy 56:224–230.

Mahut B, Delacourt C, Zerah-Lancner F, De Blic J, Harf A, Delclaux C. 2004. Increase in alveolar nitric oxide in the presence of symptoms in childhood asthma. Chest 125:1012–1018.

Mann P, Cohen AB, Finley TN, Ladman AJ. 1971. Alveolar macrophages: Structural and functional differences between non-smokers and smokers of marijuana and tobacco. Lab Investig 25:111–120.

Martinez FD, *et.al.* 1988. Parental smoking enhances bronchial responsiveness in nine-year-old children. Am Rev Respir Dis 138:518–523.

McSharry C, Banham SW, Lynch PP, Boyd G. 1984. Antibody measurement in extrinsic allergic alveolitis. Eur J Respir Dis 65:259–265.

McSharry C, Banham SW, Boyd G. 1985. Effect of cigarette smoking on the antibody response to inhaled antigens and the prevalence of extrinsic allergic alveolitis among pigeon breeders. Clin Allergy 15:487–494.

Medin NI, Osebold JW, Zee YC. 1976. A procedure for pulmonary lavage in mice. Am J Vet Res 37:237–238.

Meldrum DR, *et al.* 1998. Nitric oxide downregulates lung macrophage inflammatory cytokine production. Ann Thorac Surg 66:313–317.

Menon P, Rando RJ, Stankus RP, Salvaggio JE, Lehrer SB. 1992. Passive cigarette smoke-challenge studies: Increase in bronchial hyperreactivity. J Allergy Clin Immunol 89:560–566.

Oh SJ, Min YG, Kim JW, Lee SJ, Jarin PR. 2003. Expression of nitric oxide synthases in nasal mucosa from a mouse model of allergic rhinitis. Ann Otol Rhinol Laryngol 112:899–903.

Oryszczyn MP, Annesi-Maesano I, Charpin D, Paty E, Maccario J, Kauffmann F. 2000. Relationships of active and passive smoking to total IgE in adults of the epidemiological study of the genetics and environment of asthma, bronchial hyperresponsiveness, and atopy (EGEA). Am J Respir Crit Care Med 161:1241–1246.

Oswald IP, Gazzinelli RT, Sher A, James SL. 1992. IL10 synergizes with IL-4 and transforming growth factor-β to inhibit macrophage cytotoxic activity. J Immunol 148:3578–3582.

Patino CM, Martinez FD. 2001. Interactions between genes and environment in the development of asthma. Allergy 56:279–286.

Ronchetti R, *et al.* 1992. Enhanced allergic sensitisation related to parental smoking. Arch Dis Child 67:496–500.

Saxon A, Kurbe-Leamer M, Behle K, Max EE, Zhang K. 1991. Inhibition of human IgE production via Fc epsilon R-II stimulation results from a decrease in the mRNA for secreted but not membrane epsilon H chains. J Immunol 147:4000–4006.

Saxon A, Max EE, Diaz-Sanchez D, Zhang K. 1995. Alternative RNA of epsilon transcripts produces mRNAs encoding two membrane and four secreted IgE isoforms. Int Arch Allergy Immunol 107:45–47.

Scherer G, Richter E. 1997. Biomonitoring exposure to environmental tobacco smoke (ETS): A critical reappraisal. Hum Exp Toxicol 16:449–459.

Seymour BWP, Pinkerton KE, Friebertshauser KE, Coffman RL, Gershwin LJ. 1997. Second-Hand smoke is an adjuvant for T helper-2 responses in a murine model of allergy. J Immunol 159:6169–6175.

Seymour BWP, Friebertshauser KE, Peake JL, Pinkerton KE, Coffman RL, Gershwin LJ. 2002. Gender differences in the allergic response of mice neonatally exposed to environmental tobacco smoke. Dev Immunol 9:47–54.

Seymour BWP, Schelegle ES, Pinkerton KE, Friebertshauser KE, Peake JL, Kurup VP, Coffman RL, Gershwin LJ. 2003. Second-hand smoke increases bronchial hyperreactivity and eosinophilia in a murine model of allergic aspergillosis. Clin Dev Immunol 10:35–42.

Simoni M, et al. 2001. The Po River Delta epidemiological survey: Reference values of total serum IgE levels in a normal population sample of North Italy (8–78 yrs). Eur J Epidemiol 17:231–239.

Singh SP, et al. 2003. Prenatal cigarette smoke decreases lung cAMP and increases airway hyperresponsiveness. Am J Respir Crit Care Med 168:342–347.

Sopori M. 2002. Effects of cigarette smoke on the immune system. Nat Rev Immunol 2:372–377.

Sopori ML, Savage SM, Christner RF, Geng YM, Donaldson LA. 1993. Cigarette smoke and the immune response: Mechanism of nicotine induced immunosuppression. Adv Biosci 86:663–673.

Takenaka H, Zhang K, Diaz-Sanchez D, Tsien A, Saxon A. 1995. Enhanced human IgE production results from exposure to the aromatic hydrocarbons from diesel exhaust: Direct effects on B-cell IgE production. J Allergy Clin Immunol 95:103–115.

Teague SV, et al. 1994. A sidestream cigarette smoke generation and exposure system for environmental tobacco smoke studies. Inhal Toxicol 6:79–93.

Thomassen MJ, et al. 1997. Nitric oxide inhibits inflammatory cytokine production by human alveolar macrophages. Am J Respir Cell Mol Biol 17:279–283.

Tracey WR, et al. 1994. Immunochemical detection of inducible NO synthase in human lung. Am J Physiol 266:L722–L727.

Tredaniel J, Boffetta P, Saracci R, Hirsch A. 1994. Exposure to environmental tobacco smoke and risk of lung cancer: The epidemiological evidence. Eur Respir J 7:1877–88.

Weibel ER. 1979. Stereological methods. Vol. 1. New York: Academic press. p 415.

Witschi H, Joad JP, Pinkerton KE. 1997. The toxicology of environmental tobacco smoke. Annu Rev Pharmacol Toxicol 37:29–52.

Zhang K, Saxon A, Max EE. 1992. Two unusual forms of human immunoglobulin E encoded by alternate RNA splicing of epsilon heavy chain membrane exons. J Exp Med 176:233–243.

